# Direct vs. Video-Laryngoscopy for Intubation by Paramedics of Simulated COVID-19 Patients under Cardiopulmonary Resuscitation: A Randomized Crossover Trial

**DOI:** 10.3390/jcm10245740

**Published:** 2021-12-08

**Authors:** Leszek Gadek, Lukasz Szarpak, Lars Konge, Marek Dabrowski, Dominika Telecka-Gadek, Maciej Maslanka, Wiktoria Laura Drela, Marta Jachowicz, Lukasz Iskrzycki, Szymon Bialka, Frank William Peacock, Jacek Smereka

**Affiliations:** 1 Research Unit, Polish Society of Disaster Medicine, 05-806 Warsaw, Poland; lg5@tlen.pl (L.G.); maro.dabrowski@gmail.com (M.D.); dtelecka@gmail.com (D.T.-G.); Maciej.maslanka@uczelniamedyczna.com.pl (M.M.); 2 Instutite of Outcomes Research, Maria Sklodowska-Curie Medical Academy, 03-411 Warsaw, Poland; 3 Research Unit, Maria Sklodowska-Curie Bialystok Oncology Center, 15-026 Bialystok, Poland; 4 Copenhagen Academy for Medical Education and Simulation, Centre for HR and Education, University of Copenhagen, 2100 Copenhagen, Denmark; lars.Konge@regionh.dk; 5 Department of Medical Education, Poznan University of Medical Sciences, 60-529 Poznan, Poland; 6 Department of Emergency Medicine, Medical University of Warsaw, 02-005 Warsaw, Poland; 7 Students Research Club, Maria Sklodowska-Curie Medical Academy, 03-411 Warsaw, Poland; drelawiktorialaura@gmail.com (W.L.D.); jachowicz.marta@wp.pl (M.J.); 8 Department of Emergency Medical Service, Wroclaw Medical Univesity, 51-618 Wroclaw, Poland; lukasz.iskrzycki@interia.pl (L.I.); Jacek.Smereka@umed.wroc.pl (J.S.); 9 Department of Anaesthesiology and Intensive Care, Medical University of Silesia, 41-800 Katowice, Poland; szymon.bialka@gmail.com; 10 Henry JN Taub Department of Emergency Medicine, Baylor College of Medicine, Houston, TX 77030, USA; frankpeacock@gmail.com

**Keywords:** direct laryngoscopy, video-laryngoscopes, SARS-CoV-2, COVID-19, McGrath MAC, personal protective equipment, medical simulation

## Abstract

A safe way of securing the airway with an endotracheal tube is one of the priorities of an advanced cardiovascular life support algorithm for suspected or confirmed COVID-19 patients. The aim of this study was to compare intubation success rates (ISR) and intubation time (IT) of different laryngoscopes for simulated COVID-19 patients under cardiopulmonary resuscitation. The study was designed as a prospective, randomized, crossover trial. Fifty four active paramedics performed endotracheal intubation with a Macintosh direct laryngoscope (MAC) and McGrath videolaryngoscope (McGrath) with and without personal protective equipment (PPE). Without PPE, ISRs were 87% and 98% for MAC and McGrath, respectively (*p* = 0.32). ITs were 22.5 s (IQR: 19–26) and 19.5 s (IQR: 17–21) for MAC and McGrath, respectively (*p* = 0.005). With PPE, first-pass ISR were 30% and 89% with MAC and McGrath, respectively (*p* < 0.001). The overall success rates were 83% vs. 100% (*p* = 0.002). Median ITs were 34.0 s (IQR: 29.5–38.5) and 24.8 s (IQR: 21–29) for MAC and McGrath, respectively (*p* < 0.001). In conclusion, the McGrath videolaryngoscope appears to possess significant advantages over the Macintosh direct laryngoscope when used by paramedics in suspected or confirmed COVID-19 intubation scenarios.

## 1. Introduction

Airway management is a key element of the resuscitation process but unfortunately the first-pass success of emergency intubations are inconsistent and relatively low. Studies by Suzuki et al. [1] and Mallick et al. [2] from emergency departments found first-pass ISR using the Macintosh direct laryngoscope between 58% and 90%. Endotracheal intubation in a pre-hospital setting maybe even less effective, which is influenced by time pressure, unfavorable weather conditions, often the lack of optimal access to the patient, as well as other factors [3,4]. Breeman et al. showed that 65 nurse-staffed ambulances had first-pass ISR of 48% [5] and Arima et al. found a 75% first-pass ISR [6].

According to the guidelines of the American Heart Association (AHA), a safe way of securing the airway with an endotracheal tube is one of the priorities of an advanced cardiovascular life support cardiac arrest algorithm for suspected or confirmed COVID-19 patients [7]. This is because the new SARS-CoV-2 coronavirus is mainly transmitted by air [8,9], and the airway protection procedure itself is considered a high-risk aerosol-generating procedure (AGP) in COVID-19 [10].

It is worth noting that the use of personal protective equipment (PPE) in AGP may extend the duration of a medical procedure and reduce its effectiveness [11]. This applies to the quality of CPR performed [12,13], obtaining intravascular access [14] or the endotracheal intubation itself [15,16,17]. Therefore, it is reasonable to look for alternative methods of endotracheal intubation that will increase the effectiveness of this procedure performed in the conditions of suspected or confirmed COVID-19 patients.

The aim of this study was to compare success rates and intubation time of different laryngoscopes for COVID-19 patient intubation under cardiopulmonary resuscitation in a medical simulation condition.

## 2. Materials and Methods

The study was designed as a prospective, randomized, crossover simulation trial. The study protocol was approved by the Institutional Review Board of the Polish Society of Disaster Medicine (Approval: 12.02.2021.IRB).

Active paramedics with clinical experience with direct-laryngoscopy (minimum 10 clinical intubations) but no prior experience with McGrath MAC or any other video-laryngoscope were eligible for the study. Participants were recruited between June and September 2021 and all gave informed consent. The number of study participants was a result of voluntary enrollment; thus, a sample size calculation and power analysis were not performed.

### 2.1. Procedure

Two laryngoscopes were used for the study:

(a) McGrath video laryngoscope equipped with a size 3 Macintosh blade (McGrath);
(b) Macintosh laryngoscope (MAC) with blade no. 3, which, due to its widespread use, is the “gold standard” and a point of reference for comparing other laryngoscopes.

**Figure 1:**
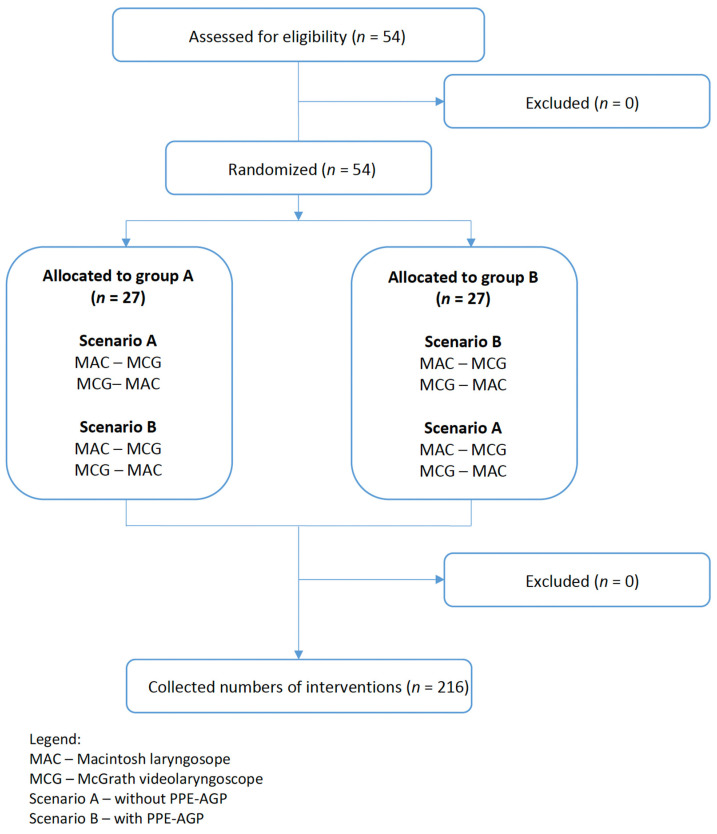
Randomization flow chart.

Each participant performed endotracheal intubations using both laryngoscopes (McGrath, MAC) in two different airway scenarios: (1) without PPE-AGP, (2) with PPE-AGP. Participants were randomized to start with either of the two scenarios, and they were further randomized to the devices for each case. In each scenario, participants had up to three attempts to intubate with each laryngoscope. A detailed randomization procedure is outlined in Figure 1.

Before starting the study, all participants took part in a 30-min training course covering the basics of endotracheal intubation based on the laryngoscopes used in the study. At the end of the theoretical training, the instructor demonstrated the correct technique of tracheal intubation with each of the laryngoscopes in question. The study participants then had 10 min to familiarize themselves with the individual laryngoscopes and to practice tracheal intubation under normal airway conditions. For this purpose, a standardized airway manikin with a regular airway (Ambu Airway Man, Ambu, Copenhagen, Denmark) was used.

A standardized high-fidelity adult simulator SimMAN 3G (Laerdal, Stavanger, Norway) was used to simulate a COVID 19 patient with cardiac arrest for the final tests. The simulator was placed on the floor level in a neutral position.

The participants of the study wore the Tychem F chemical-resistant suit, protecting against organic and inorganic chemicals in high concentrations and against particles below 1 µm in diameter (DuPont Personal Protection, Luxemburg). Additionally, the paramedics wore a protective mask with an FFP2 filter (3M Aura Disposable Respirator, FFP2, Valved, 9312+, 3M Inc., Bracknell, United Kingdom) protective goggles (MedaSEPT, Poznań, Poland), visor, and double nitrile gloves.

### 2.2. Data Collection

The participants were reminded that the “patient” is a suspected COVID-19 patient suffering cardiac arrest and needing emergency endotracheal intubation as fast as possible before each attempt. This was done to give participants a feeling of time pressure that would be present in real emergency situations.

The primary outcome was the first-pass ISR. The success of intubation was recorded when the success of the ventilation attempt was seen by the simulator’s ventilation indicators.

Secondary outcomes were overall ISR, time to intubation (IT), glottic view, and ease of intubation score. IT was defined as a time starting when the blade of the intubation device was inserted between the teeth and timing finished at the first ventilation of the lungs. Time intervals were measured by the same investigator, using a stopwatch, for all intubations. The glottic view was rated using the Cormack and Lehane grade [18]. Additionally, each participant was asked to rate the ease of intubation using a visual analogue 10-point scale (“1”-a very easy procedure to “10”—a very difficult procedure).

### 2.3. Statistical Analysis

The statistical analysis was performed with the Statistica software version 13.4EN for Windows (Tibco Inc., Tulsa, OK, USA). A *p* < 0.05 was considered statistically significant. Data are presented as number (percentage), or median (interquartile range [IQR]), as appropriate. Non-parametric tests were used for the data that did not have a normal distribution, which was tested with the Lilliefors test and the Shapiro–Wilk test. All statistical tests were two-sided. The one-way ANOVA on ranks was applied to compare the different times and to determine the statistical difference for each group (post hoc Bonferroni correction was used to counteract the problem of multiple comparisons).

## 3. Results

Fifty-four active paramedics (31% female) with a median of 5.3 years (IQR: 3–11) of experience in Emergency Medical Service Teams participated in this trial. All paramedics had previous clinical experience with direct laryngoscopy guided endotracheal intubation (median 17 (IQR: 12–35) intubations and none had any experience with any video laryngoscope.

### 3.1. Intubation without Personal Protective Equipment-Aerosol-Generating Procedure (PPE-AGP) Scenario

**Figure 2:**
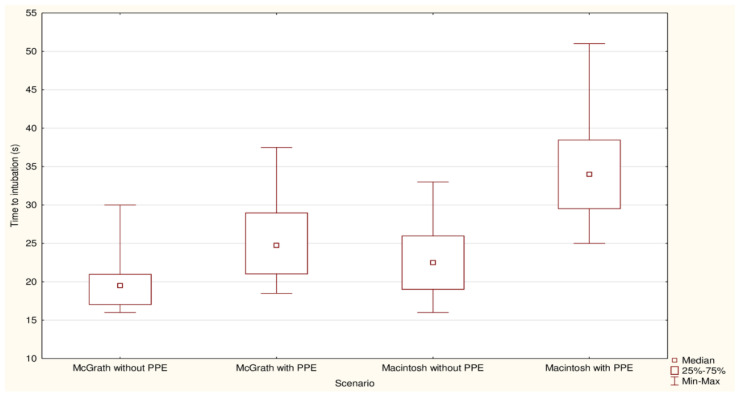
Median intubation times among McGrath and Macintosh laryngoscopes with and without PPE-AGP conditions.

**Figure 3:**
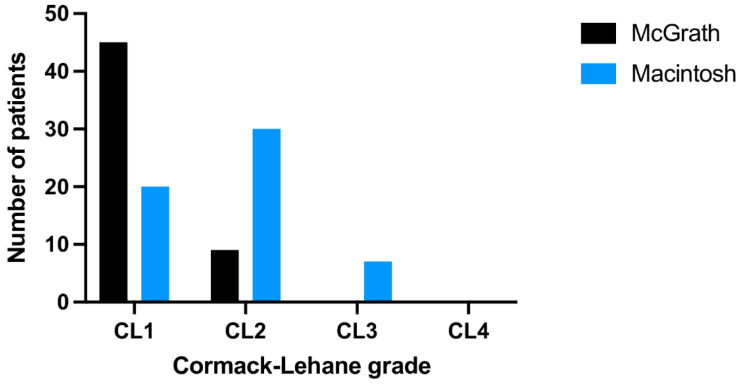
Cormack-Lehane grades in a scenario with either the McGrath or the Macintosh laryngoscope in a scenario without PPE-AGP.

First-pass ISR with McGrath was 98% compared to 87% for MAC (*p* = 0.32; Table 1). Median IT with McGrath and MAC varied and amounted to 19.5 (IQR: 17–21) vs. 22.5 (IQR: 19–26) seconds, respectively (*p* = 0.005; Figure 2). Cormack and Lehane grades 1 or 2 were observed in 100% of McGrath intubation cases and 96% in MAC group (*p* < 0.001; Figure 3). Ease of intubation in McGrath and MAC groups were 2 (IQR: 1–2) vs. 4 (IQR: 3–5) points, respectively (*p* < 0.001).

### 3.2. Intubation with PPE-AGP Scenario

**Figure 4:**
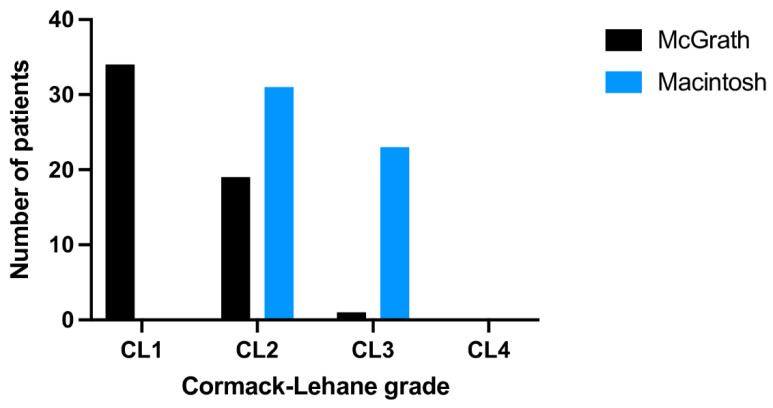
Cormack-Lehane grades in a scenario with either the McGrath or the Macintosh laryngoscope in a scenario with PPE-AGP.

The first-pass ISR with McGrath and Macintosh laryngoscopes varied and amounted to 89% vs. 30%, respectively (*p* < 0.001), while the overall ISR were 100% vs. 83% (*p* = 0.002; Table 2). Median IT with McGrath was 24.8 (IQR: 21–29) seconds and was statistically significantly shorter than with Macintosh laryngoscope—34 (IQR: 29.5–38.5) seconds (*p* < 0.001; Figure 2). Good glottis visualization (grade as C-L 1 or 2) was reported in 53 cases of the McGrath group compared to 31 cases in the Macintosh group (*p* < 0.001; Figure 4). Moreover, the procedure was rated as easier with McGrath compared with Macintosh (*p* < 0.001; Table S1).

### 3.3. Influence of PPE-AGP on Intubation with Macintosh Direct Laryngoscope

Tracheal intubation using the Macintosh direct laryngoscope in PPE-AGP conditions compared with a scenario without PPE-AGP was associated with a decrease in both the first-pass ISR (30% vs. 87%; *p* < 0.001), overall ISR (83% vs. 100%; *p* = 0.001), as well as increasing the IT (34 vs. 22.5 s; *p* < 0.001). The use of PPE-AGP was also associated with a deterioration in the visualization of the glottis, assessed according to the Cormack–Lehane scale (Figure S1; *p* < 0.001) and a more difficult-to-perform procedure, assessed on a 10-point scale (7 vs. 4 points; *p* < 0.001).

### 3.4. Influence of PPE-AGP on Intubation with McGrath Videolaryngoscope

ITs using the McGrath videolaryngoscope with and without PPE-AGP were 24.8 vs. 19.5 s (*p* < 0.001). The first-pass ISR with McGrath was 89% for a scenario with PPE-AGP and 98% for a scenario without PPE-AGP, respectively (*p* = 0.41), with 100% overall ISRs in both cases. Moreover, PPE-AGP did not affect the deterioration of glottis visualization in the McGrath videolaryngoscope (Figure S2; *p* = 0.06), but it was associated with a worsening of the difficulty in performing the procedure subjectively assessed by the study participants (3 vs. 2 points; *p* <0.001).

## 4. Discussion

In the present study, the McGrath videolaryngoscope enabled faster intubation with and without PPE-AGP conditions compared with the Macintosh laryngoscope.

The effectiveness of endotracheal intubation performed in the emergency medicine setting, as indicated by numerous publications, is insufficient [1,2]. This is especially true for intubation performed in the pre-hospital setting. In EMS teams, paramedics should be skilled in instrumental airway management. Due to the wide availability of a Macintosh laryngoscope, it is still the “gold standard” of endotracheal intubation despite the development of videolaryngoscopy [19]. However, the search for intubation methods alternative to direct laryngoscopy is one of the main areas of research in emergency medicine. According to numerous studies, videolaryngoscopy, compared to direct laryngoscopy, shows a faster learning curve [20,21], as well as offering better glottis visibility and higher intubation efficiency, especially for intubation in difficult conditions (i.e., difficult airway conditions or difficult access to patients airway) [22,23].

Endotracheal intubation under normal airway conditions—when medical personnel are not wearing PPE-AGP—showed no statistically significant differences between the McGrath and Macintosh laryngoscope in terms of both first-pass ISR (*p* = 0.32) and overall ISR (*p* = 1.0), however, videolaryngoscopy provided shorter procedure duration (*p* = 0.005) as well as better glottis visualization (*p* < 0.001) In normal airway management conditions, videolaryngoscopy does not show advantages to direct laryngoscopy also in other studies [24,25].

The current COVID-19 pandemic has forced medical personnel to perform a high-risk aerosol-generating procedure using protective suits [26]. This is of particular importance in the context of emergency medical service teams, where each patient should be treated as potentially infectious. However, the use of PPE-AGP, as indicated by some authors, may reduce the effectiveness of individual procedures, referring both to the increase in procedure duration and decrease in its efficiency [27]. This is dictated by the discomfort of medical personnel who are wearing PPE-AGP, the reduced sensitivity resulting from the use of double gloves, and the often-reduced visibility through the use of safety glasses and visors (which tend to fog up) [28,29,30].

In our study, the use of PPE-AGP decreased the success rate of the first intubation attempt with a Macintosh direct laryngoscope (*p* < 0.001) as well as increasing the duration of the procedure (*p* < 0.001) for a McGrath videolaryngoscope and Macintosh direct laryngoscope. In contrast, in a study by Taylor et al. emergency medicine residents and pre-hospital providers were enrolled to either intubation with a direct- or a video-laryngoscope in a cadaveric model while wearing level C PPE or without PPE [30]. In the above study, for direct laryngoscopy first-pass ISR was 100% without PPE and 79% while wearing PPE (*p* = 0.11), while VL had a 100% success rate without PPE and 74% in PPE (*p* = 0.046).

It is worth noting, however, that unlike our study (where paramedics alone participated), the Taylor et al. study included mostly emergency medicine residents. In the study by Pantazopoulos et al. [31] 70 inexperienced physicians showed that first-pass ISR and IT with the McGrath MAC video laryngoscope was not affected by PPE donning which is also confirmed by the results of our study. However, in the study by Pantazopoulos et al. as in our study, a negative effect of intubation with the PPE-AGP suit was observed on the first-pass ISR, the total ISR as well as the IT performed with the Macintosh direct laryngoscope. The negative effect of PPE-AGP on the effectiveness of direct laryngoscopy is also indicated by the meta-analysis by Ludwin et al. [32] as well Caglar et al. Their study showed shorter intubation times using videolaryngoscopy compared to direct laryngoscopy when medical personnel were wearing a level C suite [33].

Another important element affecting the efficacy and duration of intubation is the degree of glottis visualization during the intubation procedure. Compared to Macintosh laryngoscopy, videolaryngoscopy enables better visualization of the glottis, particularly in patients with difficult airways or when optimal access to the airway is not possible. In such situations, videolaryngoscopes offer an advantage over intubation based on direct laryngoscopy due to image transmission from the distal end of the laryngoscope blade. In patients with suspected or confirmed COVID-19, endotracheal intubation should be performed as soon as possible without bending over the patient because of the risk of virus transmission during the procedure [34]. In our study, better visualization of the glottis was observed with the McGrath than with the Macintosh laryngoscope. This difference was statistically significant. Glottic visualization with and without PPE-AGP was different for Macintosh direct laryngoscopy (*p* < 0.001). In contrast, it did not show a statistically significant difference for intubation with a McGrath videolaryngoscope.

### Strengths and Limitations

There are some shortcomings of the present study that need to be addressed. First, this is a manikin rather than a clinical study. Such an action was deliberate, as the simulation allows for full standardization of medical procedures without the risk of complications for the patient as well as the risk of infection of medical personnel in patients with suspected or confirmed COVID-19 [35,36]. It is worth noting that the study used high-fidelity medical simulation. Another limitation is the lack of blinding the study, however, due to the nature of the study and the procedures performed, it is impossible to blind the study at the stage of its performance; nonetheless, the results were blinded before the stage of statistical analysis. The third limitation is the fact that the study was performed among paramedics who had no previous experience in the field of videolaryngoscopy; however, this action was also purposeful due to the equal level of skills of the study participants. Another potential limitation is that we chose to use the Cormack and Lehane grade tool to assess glottic visualization. However, this scale was used due to its universality. Another limitation is that paramedics taking part in our study had very limited exposure to endotracheal intubation in the prehospital settings. EMS systems differ in a number of characteristics, including but not limited to the range of skills, experience, and qualifications of paramedics, including endotracheal intubation [37,38]. In our study, we analyzed paramedics with relatively limited experience in endotracheal intubation by direct laryngoscopy. It should be noted, however, that the learning curve is much more favorable for endotracheal intubation with videolaryngoscopes than when direct laryngoscopy is used. The results obtained may be particularly relevant for some EMS systems in which rescuers have similar experience and range of skills.

Beyond limitations, the study also has strengths such as the randomized cross-over nature of the study and use of a suspected or confirmed COVID-19 patient intubation scenario.

## 5. Conclusions

In conclusion, the McGrath videolaryngoscope appears to possess advantages over the Macintosh direct laryngoscope when used by paramedics in suspected or confirmed COVID-19 intubation scenarios.

## Figures and Tables

**Table 1:** Intubation with McGrath and Macintosh laryngoscopes without personal protective equipment-aerosol-generating procedure (PPE-AGP) scenario.

Parameter	McGrath	MAC	*p* Value
Overall success rate, *n* (%)	54 (100%)	54 (100%)	1.0
Time to intubate (s), median (IQR)	19.5 (17–21)	22.5 (19–26)	0.005
Number of intubation attempts, n(%)			0.32
1	53 (98%)	47 (87%)	
2	1 (2%)	7 (13%)	
3	-	-	
Cormack & Lehane grade			<0.001
1	45 (83%)	20 (37%)	
2	9 (17%)	30 (56%)	
3	-	4 (7%)	
4	-	-	
Ease of intubation (1–10), median (IQR)	2 (1–2)	4 (3–5)	<0.001

**Table 2:** Intubation with McGrath and Macintosh laryngoscopes with PPE-AGP scenario.

Parameter	McGrath	MAC	*p* Value
Overall success rate, *n* (%)	54 (100%)	45 (83%)	0.002
Time to intubate (s), median (IQR)	24.8 (21–29)	34 (29.5–38.5)	<0.001
Number of intubation attempts, n (%)			<0.001
1	48 (89%)	16 (30%)	
2	6 (11%)	21 (39%)	
3	-	17 (31%)	
Cormack & Lehane grade			<0.001
1	34 (63%)	-	
2	19 (35%)	31 (57%)	
3	1 (2%)	23 (43%)	
4	-	-	
Ease of intubation (1–10), median (IQR)	3 (2–4)	7 (6–8)	<0.001

## Data Availability

The data will be available from the corresponding author on request.

## Supplementary Materials

The following are available online at https://www.mdpi.com/article/10.3390/jcm10245740/s1, Table S1. Multiple comparison *p* values, Figure S1: Cormack-Lehane grades in intubation with Macintosh laryngoscope with and without PPE-AGP scenarios, Figure S2: Cormack-Lehane grades in intubation with McGrath laryngoscope with and without PPE-AGP scenarios.
