# Supplementary material for: Direct vs. Video-Laryngoscopy for Intubation by Paramedics of Simulated COVID-19 Patients under Cardiopulmonary Resuscitation: A Randomized Crossover Trial
Source: J Clin Med. 2021 Dec 8;10(24):5740. doi: 10.3390/jcm10245740 (PMC8707195; doi:10.3390/jcm10245740)
Supplement: Supplementary file 1 [file jcm-10-05740-s001.zip › jcm-1457501-supplementary.pdf]

Table S1. Multiple comparison *p* values.

	**McGrath without PPE**	**McGrath with PPE**	**MAC without PPE**	**MAC with PPE**
Time to intubation				
McGrath without PPE	-	<0.001	0.13	<0.001
McGrath with PPE	<0.001	-	0.07	<0.001
MAC without PPE	0.13	0.07	-	<0.001
MAC with PPE	<0.001	<0.001	<0.001	-
The first-pass intubation success rate				
McGrath without PPE	-	1.0	1.0	<0.001
McGrath with PPE	1.0	-	1.0	<0.001
MAC without PPE	1.0	1.0	-	<0.001
MAC with PPE	<0.001	<0.001	<0.001	-
Overall success rate				
McGrath without PPE	-	1.0	1.0	0.807
McGrath with PPE	1.0	-	1.0	0.807
MAC without PPE	1.0	1.0	-	0.807
MAC with PPE	0.81	0.81	0.81	-
Glottic visualization				
McGrath without PPE	-	0.56	<0.001	<0.001
McGrath with PPE	0.56	-	0.13	<0.001
MAC without PPE	<0.001	0.13	-	<0.001
MAC with PPE	<0.001	<0.001	<0.001	-
Ease of intubation				
McGrath without PPE	-	< 0.001	< 0.001	< 0.001
McGrath with PPE	< 0.001	-	0.76	< 0.001
MAC without PPE	< 0.001	0.76	-	< 0.001
MAC with PPE	< 0.001	< 0.001	< 0.001	-

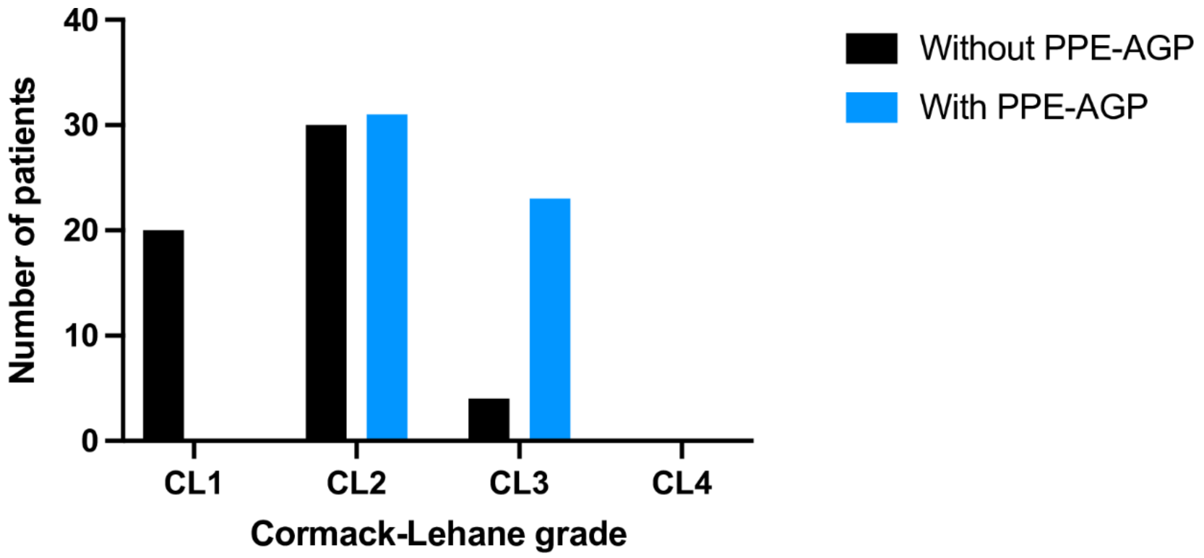

Figure S1. Cormack-Lehane grades in intubation with Macintosh laryngoscope with and without PPE-AGP scenarios.

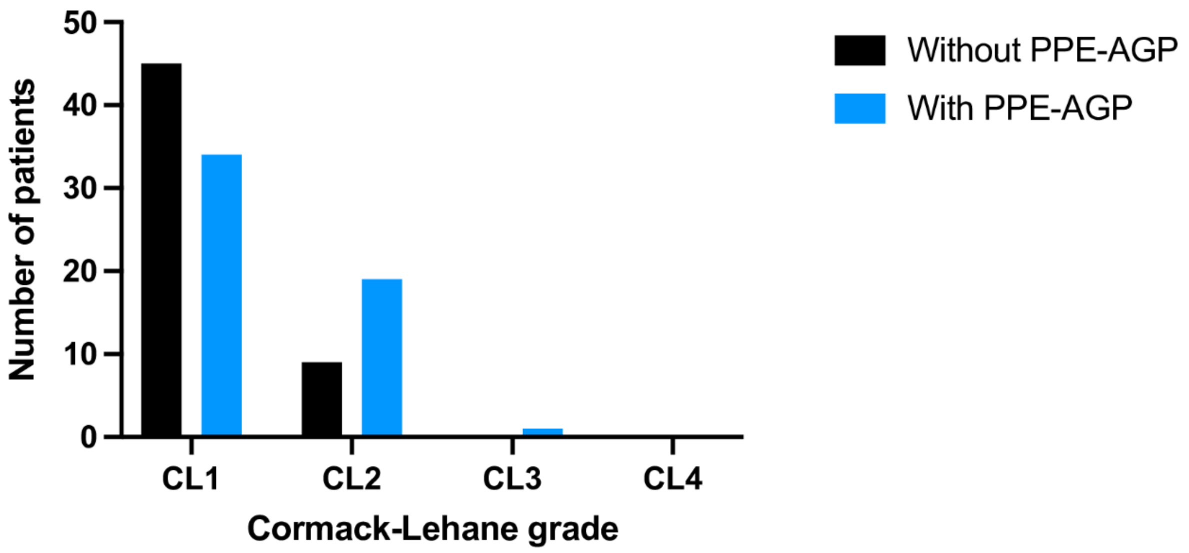

Figure S2. Cormack-Lehane grades in intubation with McGrath laryngoscope with and without PPE-AGP scenarios.
